# What are the chances? Clinician scientist` career pathways in Germany

**DOI:** 10.1186/s12909-023-04584-8

**Published:** 2023-09-07

**Authors:** Barbara Hendriks, Martin Reinhart

**Affiliations:** Robert K. Merton Center for Science Studies (RMZ) at Humboldt-Universität zu Berlin, Berlin, Germany

**Keywords:** Translational medicine, Clinician scientists, Clinician scientist programs, Research training, Translational training

## Abstract

**Background:**

Germany faces a lack of clinician scientists. This problem is widely acknowledged, not just in Germany, as clinician scientists are crucial for medical translation and innovation: trained in medical practice and research they are capable of translating scientific problems into clinical application and vice versa, clinical problems into research. The implementation of nationwide clinician scientist programs (CSPs) in Germany is supposed to solve the lack of trained clinician scientists and, as consequence, to improve the translational relationship between biomedical research and clinical practice. Against the backdrop of an increasing number of CSPs, our study provides early insights into their effectiveness with a focus on what it means to become a clinician scientist and to establish a subsequent career path as a clinician scientist in Germany.

**Methods:**

During a research project that was conducted from 2020 to 2023 and funded by the German Federal Ministry of Education and Research, we studied thirteen CSPs. We developed a qualitative questionnaire and interviewed 36 clinician scientists in training, their program supervisors, as well as policy stakeholders. The goal of the interviews was to identify the key obstacles in establishing a career path for clinician scientists in Germany.

**Results:**

We found three types of challenges for establishing and ensuring long term career paths for clinician scientists: First, local working conditions need to allow for clinician scientists to create and perform tasks that combine research, teaching, patient care and translation synergistically. Protection from the urgency of patient care and from metrics-based performance measures both in the clinic and in research seem key here. Second, a stable career path requires new target positions besides clinic management and senior residency. Third, there is a need for cultural change within university medicine that recognizes and rewards new translation-focused practices.

**Conclusion:**

We find that CSPs improve working conditions for the duration of the program and provide protected time for doing research. After the programs, however, the career paths remain unstable, mainly due to a lack of target positions for clinician scientists. CSPs support the initial development of the clinician scientist’ role, but not in a sustainable way, because the separation of research and patient care is stabilized on an institutional and systemic level. The tasks clinician scientists perform in research remain separate from patient care and teaching, thus, limiting their translational potential. In order to remain a clinician scientist within this differentiated system of university medicine, clinician scientists have to do a significant amount of extra work.

**Supplementary Information:**

The online version contains supplementary material available at 10.1186/s12909-023-04584-8.

## Background

Increasing the number of clinician scientists has become a national science policy strategy in Germany. The German Research Council (DFG) formulated in 2015 [4] specific recommendations for the "establishment of integrated research and continuing education programs for clinician scientists in parallel with residency training” and a year later The German Science and Humanities Council ([16]: 29) considered it appropriate that, “five to eight percent of physician doctors who complete their residency training at university (approx. 2,300 persons per year) as clinician scientists (approx. 110 to 180 physicians per year)”. As physicians who are solidly trained in (bio)medical and clinical research, clinician scientists bridge the innovation gap that is caused by the separation of biomedical research and medical practice. In accordance, various clinician scientist funding schemes were implemented to increase the number of clinician scientists. Today, 37 of the 38 university hospitals in Germany run a clinician scientist program (CSP), often externally funded through public programs. No specified framework for clinician scientist training exists, yet, but there are recommendations for core elements: “protected research time” in order to conduct a “research project”, “mentoring” and an “advanced training curriculum” (DFG 2015) and the DFG recommends the BIH Charité (Junior) Clinician Scientist Program as best practice. Programs usually distinguish between junior-clinician scientist programs, which target physicians in the second and third year of their residency and regular clinician scientist programs for those physicians who are in the last two to three years of their residency training. More recently the “Advanced-Clinician Scientist Program” has been introduced for already trained medical specialists. The arising variety of CSPs illustrate the impact of political measures: clinician scientists recently have become a key element of doctoral training within university medicine in Germany.

With the nationwide growth of CSPs, we want to know, to what extent clinician scientists are integrated into the system of German university medicine. How are career prospects of (trained) clinician scientists? We think, now it is time to clarify these questions, because, if clinician scientists are increasingly being trained, adequate career paths are needed for this emerging profession in which they can permanently exercise their new role within university medicine [11]. Otherwise, (publicly) funded training of clinician scientists would not meet its primary goal of permanently increasing the number of clinician scientists and also not the secondary, systemic goal of improving translation.

Despite their global importance in science policy [15], there is little empirical research on CSPs in Germany (exceptions are: Bossé et al. [3]; Hendriks et al. [10]). This stands in contrast to the situation in Canada (Twa et al. [14]; Bookey-Basset et al. 2022 [2]; Pietrobon et al. [13]), the USA (Eshel and Chivukula [9]), Asia (Yoon et al. [19]) or Australia (Eley et al. [8]; Eley et al. [7]), where everyday challenges of clinician scientist pathways and training are widely discussed.

In Germany, there is a wealth of recommendations and discussions on how to design CSPs (see DFG [4]; Baum et al. [1]; Wissenschaftsrat [16–18]), but still very little research on how they work. To address this shortcoming, we provide insights from a research project that explores career pathways of clinician scientists in Germany.

## Methods

To analyze clinician scientists career paths in more depth, we conducted 36 expert interviews in the context of 13 different CSPs in Germany. We invited all existing 32 CSPs to our study from which 13 CSPs responded and participated within study deadline. The goal was to assemble a comprehensive as well as heterogeneous sample of CSPs including CSPs that vary in size (number of clinician scientists, sub-programs, and scope of disciplines), in time-span since inception of the programs (more than 10 years of experience vs. recently established) and in relation to the size of the hospital (number of beds).

We conducted the interviews in the years from 2020 to early 2023. Since our project was strongly influenced by the Covid-19-pandemic and visitor rules in university hospitals were particularly regulated and clinics partially not accessible for third parties or researchers, we conducted all interviews digitally face-to-face. Increasing work load due to the pandemic has made interviewing considerably more difficult because the clinically active physicians had little to no capacity to do so. Clinician scientists reported that they had to take a partial break from their “protected” research time and were assigned to the clinic full time. In this respect, the video calls were helpful because they reduced the effort required for physicians to participate in the study.

We interviewed experts from the CSP-management and from politics (*n* = 24) as well as clinician scientists in training (*n* = 12) in order to capture perspectives from both management and clinician scientists in training (for detailed distribution see appendix). All selected interview participants were invited to an interview by a formal letter and participants were provided written consent, which has documented the aim of our study and how data is processed and anonymized. The “Medizinische Fakultätentag”, an organization representing interests of 38 medical faculties in Germany as well as the Berlin Institute of Health supported our study with a recommendation letter. All interviews were structured by interview guidelines, recorded and transcribed and then analyzed and coded completely with MAXQDA to ensure comparability of interview statements. Coding and analysis was done according to qualitative content analysis [12].

In order to answer our main research question “To what extent are clinician scientists integrated into the system of German university medicine?”, we draw on answers of three questions posed in the guided interview: (1) What motivates you/clinician scientists to participate in the CSP? (2) Beyond professorship, what target positions can you look forward to as a clinician scientist/what target positions can clinician scientists look forward to? (3) What career requirements must be met to take such a position? We coded these questions inductively, i.e. not hypothesis-driven. Taken together, these three questions provide insights into the extent of integration of clinician scientists within university medicine.

## Findings

### Improved working conditions

What motivates clinician scientists to participate in a CSP? Why is the clinician scientist career path worth pursuing? We identified four key motives in the interviews, which are mentioned with varying frequency (see Table 1): (1) The possibility to conduct research (46%), (2) to participate in a prestigious and thus career-promoting program (23%), (3) to end research outside of regular working hours (so-called “Feierabendforschung”) (16%) and (4) to profit from better research conditions (15%). See Table 1 for representing statements for each category.

**Table 1:** Representative quotes for what motivates clinician scientists to pursue a CSP

Major theme	Representative quotes	Freq
To conduct research	*“The motivation for that [CSP application] was to finally do research again” (clinician scientist, CSP 3). “And then I realized for myself that I do have a certain talent for research. And that, on the other hand, I simply find it interesting and beautiful. And that I find it a fulfilling addition to my daily clinical routine” (Clinician scientist, CSP 6)*	46%
To participate in a prestigious program	*“At the university, for example, it is relatively prestigious to be accepted into such a program. So, it certainly plays a role in your further career” (Clinician scientist, CSP 4)* *“For example, for third-party funding applications that we have just submitted. When it comes to keeping your CV in good shape, I have the feeling that being in a clinician scientist program is definitely an advantage” (Clinician scientist, CSP 4)*	23%
To end after-work research	*“When you're assigned as a doctor, you have scheduled working hours, early shift, 8 am to 5, 6 p.m., late shift 1 to 10 p.m., that's the way it is. Then, on weekends, night duty or 20-h shifts. Of course, it doesn't stop there, so the medical work is then connected with overtime depending on the patient volume, the emergencies and so on. This means that if I were to outline a classic day, I would start at 8 o'clock on a ward and then leave at 6 or 7 p.m., in a good case. That is, I would say, realistic. And if you still want to do research, then you would do it in the evening” (Clinician scientist, CSP 1)* *“And that completely tore me apart mentally and physically, because the activities of the clinic and the laboratory are so different and both are very intensive in terms of time, that it is difficult to somehow reconcile them. And you can't really do either one or the other well or satisfactorily for your own requirements, so I decided that I had to do research full time now in order to advance these projects further and more intensively” (Clinician scientist, CSP 1)*	16%
To profit from better research conditions	*“In addition, there is also third-party funding. So regardless of the 50 percent funding, you still get third-party funding *via* the research project, which is of course also lucrative, especially as follow-up funding” (Clinician scientist, CSP 6)*	15%

The opportunity to do research is one of the main factors for pursuing a clinician scientist career path. This finding follows previous study results [10], since performing research in the context of full-time clinical care work is hardly possible in German university medicine – or only possible with extraordinary personal sacrifice.

*Do we want to do clinical research? Do we want to do translational clinical research that is relevant and corresponds to the current state of knowledge in research? If so, we need people who have time for that. There is no way that this can be done in parallel with a full-time clinical position (CSP manager, CSP 6).*

Integrating research as part of a physician's profile within university medicine is a widely acknowledged challenge, and just the attempt at facing this challenge, is seen positively by many of the respondents. While the CSPs improve working conditions, such as allotting time for research within regular working hours (“protected research time”), challenges remain and relate primarily to clinical tasks taking over the protected time for research. This is problematic for two reasons: first, research time is financed from external funds, i.e., it does not come from the budget that is earmarked for patient care, which leads to intraorganizational pressure to prioritize clinic over protected research time. Second, and as a consequence, it promotes after-hours research, i.e. unpaid overtime, which works against the main motivation for joining a CSP.

### Target positions

Adequate target positions for clinician scientists are highly relevant and science policy has been dealing with this issue for some time now. Without clear target positions, the question arises as to whether such broad-based funding of clinician scientists makes sense. Our data shows that clinician scientists can perform their dual role as physician and researcher relatively well during the clinician scientist training, with some restrictions (see above). However, program funding is limited and thus also the career as a clinician scientist. Since funding episodes for clinician scientists are temporary (generally two to three years), clinician scientists state that they need already to start thinking during the grant period about what will happen afterwards and how to maintain their dual role.

*Fixed-term research funding. And you have to, as I said, reapply again and again. And it is basically a purely temporary thing. And basically, my research leave started with the fact that it was immediately said, "Mr. Müller [pseudonym], think carefully when you're back in the clinic in twelve months, how your research project will continue." And basically, in the first month you're already thinking: How do I organize the project so that after twelve months I'm back in the clinic? And that's sort of the essence of the story. Of course, these twelve months off [from clinical duties] are worth a lot, because without them it wouldn't work at all. But, of course, they don't solve the problem (Clinician scientist, CSP 6).*

To provide clinician scientists with long-term career options, science policy, but also the majority of interviewed CSP-managers, are dealing with different and rather vague ideas of how to secure clinician scientists permanently within university medicine.

*The idea would be, you make yourself an overall concept, where you say, okay, I want to promote clinician scientists now, and promote them in such a way that they can actually live this without giving up their life, not being able to start a family, and then they become a good clinician scientist. And that's kind of not the case right now (CSP manager, CSP 5).*

The fact that many of the clinician scientists return to the clinic full time after their CSP-graduation has led to the implementation of the so-called ‘Advanced-clinician scientist program’. For those clinician scientists, who want to continue their dual role after CSP-graduation, there is an opportunity to move into the Advanced-program after completing their residency training. However, this additional funding episode only prolongs the state of career uncertainty and does not, so far, solve the problem regarding adequate long-term target positions for clinician scientists.

Next to Advanced-CSPs, different (interviewed) policy stakeholders and certain CSP-managers discuss different clinician scientist-types as a defining foil for possible clinician scientist target positions within the clinic. For example, there should be long-term clinician scientist positions, where clinician scientists may either perform 50 percent of their working time in research (50–50-model) or 25 percent of their working time (the 75–25-model). The idea behind this individual modeling is that target positions should adapt to the needs of potential clinician scientist job holders. However, according to our interview data, it appears that this modeling may not be the ideal solution for clinician scientist target positions within the clinic in general. Current target positions, such as the senior physician, which is named by many interviewed experts as a realistic target position for clinician scientists next to professorship, do not allow any room for research, since these positions are already limited by their formal tasks such as personnel management, administration and patient care. Taking a portion of these positions for research would only exacerbate the existing problem of clinical staff shortage. Some experts criticize that protected research time of clinician scientists cannot be replaced adequately and having a clinician scientist on the ward also means for clinic managers to lose a percentage of staff for clinical obligations.

*CSPs first of all lead to the fact that there are even fewer people in the clinic. And the time that clinician scientists are released for research, it can't be fully replaced in the clinic. That means we don't have adequate replacements for these people on the ward (CSP manager, CSP 6).*

Currently, there are no final nor ideal target positions for clinician scientists (yet), even though the question is becoming more urgent, not only because the number of clinician scientists is increasing, but also because clinician scientists are embarking on a new career path, which in turn comes with specific challenges.

### Career requirements

What requirements must be met to take on a career as a clinician scientist? CSPs intend to improve working and research conditions for clinician scientists in university medicine while simultaneously promoting translation. To this end, institutional resources are made available (e.g. through internal or external research funding). At first glance, it appears that the reorganization of clinical towards translational work results in improvements at the individual level, because physicians are temporarily relieved from patient care in order to perform basic or clinical research. This is generally felt to be an improvement of working conditions compared to full time clinical work. However, besides the fact that protected research time is taken over by the clinic, our interviews point to further unintended side effects that need to be looked at more closely.

**Fig. 1:**
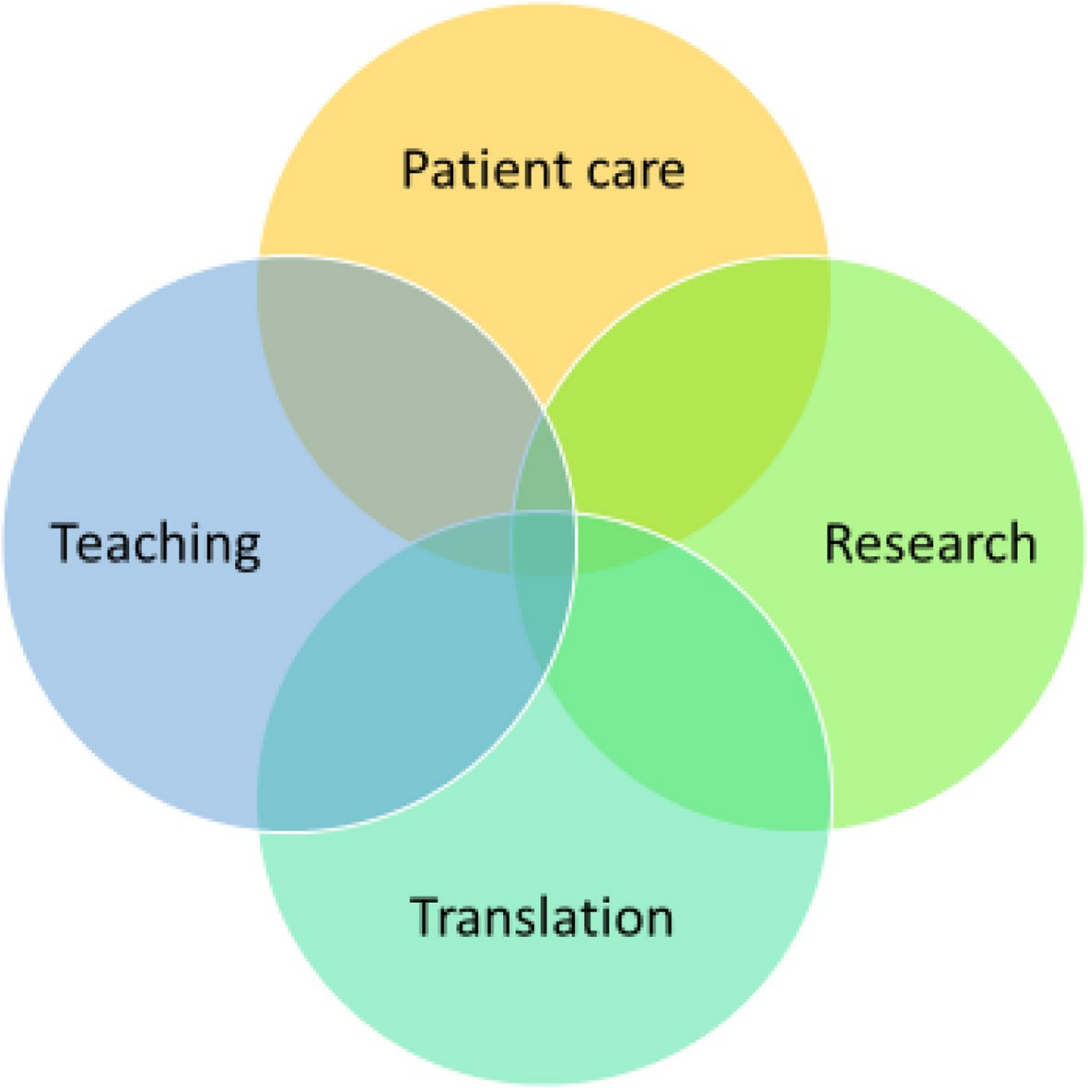
The clinician scientist quadriga

We find that the quadriga of patient care (see Fig. 1), research, teaching and translation lacks integration, as each aspect is evaluated separately. As a result, `doing translation´ becomes an additional selection mechanism in the course of a single career. Translation in itself is a process that focuses on optimizing a longer-term course. In practice, this process is broken down into its individual parts to be evaluated through specific evaluation indicators. Accordingly, evaluation criteria that are introduced to account for translation remain rather narrow and, in addition, are not necessarily compatible with established indicators for research (publication output, h-index, etc.) or patient care (bedtime for patients). In this respect, clinician scientists have to accommodate different evaluation criteria in the context of their career.

*You have three years. You get to grips with a research project like this. And it’s mostly about: Yes, how do I get a publication with as many impact points as possible and as quickly as possible? And how do I, as efficiently as possible, do as many projects at the same time so that I get to my number of H-Index points for the habilitation? That it is really about the content, that I would sometimes really doubt, I must say. It's just a lot of means to an end (Clinician scientist, CSP 1).*

Thereby, and this is the second problem, the definition of "successful translational criteria and indicators" is still fluid, as no indicators have proven to be successful in the long run, which is due to the fact that the field of translation is an emerging field and therefore turns out to be very dynamic. The negotiation of what constitutes adequate indicators of successful translation (e.g. patents, spin-offs, papers, etc.) becomes a central area of uncertainty at the level of individual clinician scientists.

*So, I came with a very good publication score, with many impact points, with a good PHD, excellent grades. And that didn't lead to the feeling that I had to be given special support. It would just be: "Yes, you have previous experience. You can handle it yourself (Clinician scientist, CSP 9).*

While the specialist catalog specifies how many operations must be performed to pass the specialist examination, it remains open as to what counts as “successfully passed” on the research and translation side. As a consequence, the principle of excellence is applied: the more (publication, third-party funding, translational projects, patents, etc.) the better.

*Of course, research is important and, of course, I want to do my translational project, but I have to become a surgeon first and then everything else will come. That’s why I tried to complete everything in parallel: to push my operational quota [number of surgical interventions] forward, on the one hand, and to push the literature work forward, on the other hand. But, of course, my personal time has suffered from it (Clinician scientist, CSP 2).*

The clinician scientists have no choice but to compensate for this uncertainty regarding the definition of "success" in research and translation with additional work in order to develop a career portfolio that reflects a spectrum as broad as possible.

## Discussion

We find that clinician scientists currently face the challenges of overtime to perform research and uncertain career paths. Clinician scientists benefit from better working conditions during the program; even despite the observed non-intended side effects. CSPs create better working conditions in the clinic, but being a clinician scientist is and remains challenging in the context of German university medicine. Future perspectives for clinician scientists are limited to the degree that the positive effects of CSPs on working conditions are significantly diminished. In particular, the lack of perspectives and stable careers lead to insecurity, which is compensated for by additional research work at the individual level. One major aim of CSPs in Germany is to reduce or prevent so called “Feierabendforschung [after-hours research]” [6], which we find is often not achieved in daily practice. In turn, the contractually stipulated research time is often substituted by work in the clinic. Whether the extra work leads to an improvement of the (future) career situation remains an open question. What improves the most are prospects for clinician scientists that seek a traditional career in research, for which traditional criteria of excellence are central. This must be seen as an unintended consequence of CSPs, as their primary institutional goal is to facilitate "compromises" between different fields of expertise such as patient care, research and teaching and move away from a singular focus on scientific excellence.

The lack of an indicator for successful translation or translational research exacerbates this problem by not providing coherent and much needed guidance for action by clinician scientists. The regime of evaluation for residency training ensures basic professional standards (pass or fail) while the evaluation of translational success depends on the criterion of scientific excellence, which follows the principle of "the more the better". This principle leads to constant extra work with the possibility of an excessive burden on individuals. It is also questionable whether this mode is actually beneficial for translation or translational research. In that regard, Dirnagl et al. [5] are discussing “external validity as a relevant modulator of result reproducibility and translatability for translation”.

For a successful, i.e. long-term career as a clinician scientist in Germany, current job profiles within university medicine have to become more flexible. This is exactly what is already happening within CSPs, by redistributing resources and positions to generate (protected) research time for clinically active physicians. However, redistribution succeeds only partially, because research is still being taken over by clinical work, despite CSP-funding, i.e. clinical care is cross-financed by public funds for research. The reasons for this are both the acute funding shortage of patient care and the unclear career requirements for clinician scientists. Assuming that the first problem – despite various ongoing discussions about it – will not be solved in the near future, the attempt would be to address the second problem (more strongly): To create a clear requirement profile for clinician scientists, which are based on clear, comprehensible indicators, similar to the catalog of medical specialists. Such profiles, however, will have to move beyond protecting a certain percentage of the work week for research and will have to define professional tasks that integrate research, patient care, teaching, and translation. Providing clear and also viable career paths for clinician scientists is also discussed in the qualitative study by [19] conducted in Singapore. Furthermore, Williams et al. [15], taking a global perspective, argue that being a clinician scientist comes with a certain risk taking, because “many may have chosen medicine as a path with job security”, which is given up with a clinician scientist career.

Our results provide an in-depth, yet limited, insight into the work reality of CSPs. Due to the methodology, our study does not claim to be representative in a quantitative sense. Additional studies that provide a broad view, e.g. through surveys, would be useful from here.

## Conclusion

We find that clinician scientists currently face the challenges of working overtime to perform research and uncertain career paths. To create a clear requirement profile for clinician scientists that integrate research, patient care, teaching, and translation could contribute to improvement and seems necessary to support the cultural change that CSPs are purported to facilitate.

### Supplementary Information

**Additional file 1.** Interview questionnaire: KeTAK project^1^.

**Additional file 2: S table 1.** Studied clinician scientist programs in Germany. To ensure anonymization, Table 1 is randomized. **S table 2.** Distribution of expert interviews per institution and level.

## Data Availability

The datasets generated and analyzed during the current study are not publicly available due to ethical reasons [participants are assured that only anonymized data extracts, but never entire transcripts, will be passed on to third parties] but anonymized data excerpts are available from the corresponding author on reasonable request.
