# Supplementary material for: What are the chances? Clinician scientist` career pathways in Germany
Source: BMC Med Educ. 2023 Sep 7;23:642. doi: 10.1186/s12909-023-04584-8 (PMC10486072; doi:10.1186/s12909-023-04584-8)
Supplement: Supplementary file 1 — Additional file 1. Interview questionnaire: KeTAK project1. [file 12909_2023_4584_MOESM1_ESM.docx]

# Interview questionnaire: KeTAK project^1^

^1^The original questionnaire is written in German. This is a translated version.

**INTRODUCTION TO THE PERSON (5 MINUTES)**

Today I would like to talk to you about *concrete CSP*. However, before we go into detail, I would like to know something about you as a person and your work here at *concrete CSP *.

- Since when have you been working for *concrete CSP* and which tasks do you perform here?

**HISTORY OF THE DEVELOPMENT OF THE ORGANIZATIONAL STRUCTURE (10 MINUTES)**

Turning now to the *concrete CSP*....

- Can you give me an overview of the genesis of the *concrete CSP* in *location*?
  - What were the biggest milestones in the creation of the *concrete CSP*?
  - What were the (strategic) goals in the creation of the * concrete CSP* in *location*?
  - What were particular challenges you faced in establishing the *concrete CSP*?

Which networks and cooperations were of central importance for the successful establishment of the *concrete CSP* and how did they come about?

- In your opinion, what are the concrete central actors and stakeholders that were involved in the establishment of *concrete CSP* and are involved in its further development?
- With which expectations was *concrete CSP*confronted by these stakeholders and how did you deal with them?
  - To what extent have expectations changed over time?

REIMAGINING RESEARCH AND PATIENT CARE (10 MINUTES)

The primary purpose of the CSP is to ensure the translation of scientific knowledge.

- To what translational needs does the *concrete CSP* respond to in particular?
  - What kind of translational understanding underlies the *concrete CSP* and how is it concretely implemented?
  - Which general translation-specific objectives are pursued at your location?
  - Which theoretical models, guidelines or policy documents do you generally follow when it comes to translation?
- How does translation work in *concrete CSP*?
  - Which other areas/departments/organizational units at the *location* are also concerned with the topic of translation and are linked to the work of the *concrete CSP*?
- How do they ensure the quality of their translational research?
  - How would you describe "best practice" translational medicine?
  - How do you document your successes to outsiders and co-funders?

**PROFESSIONAL DOMINANCE (10 MINUTES):**

In addition to ensuring successful translation, CSP should also contribute to improving the working and career conditions of physicians who wish to work in research and clinical practice. In order to be able to classify this contribution, however, we believe that it is necessary to take a look at what has changed at all for physicians in terms of job profile and career, or identify where central problems lie.

- In this context, we would first like to hear your assessment of how the job profile and careers in medicine have changed over the last 10 years, especially for those working at university hospitals?
  - What do you think is the role of Clinician Scientist Programs such as *specifically CSP* in the context of the changes you name?
- To what extent does *concrete CSP* influence physician independence and decision-making competence in research and patient treatment?
- To what extent does *concrete CSP* fundamentally change the way medical hierarchies and cooperation function in the context of university medicine?
  - To what extent does *concrete CSP* influence the careers and target positions of young physicians, especially those who aspire to a career in research in addition to clinical?
  - What do you think is needed to ensure research-oriented careers are sustained?
  - Do you think clinician scientists increasingly represent a distinct professional group?
- Can you see the clinician scientist becoming established as a realistic career in the long term?
  - What role do new funding instruments such as the Advanced-Clinician scientist play in establishing new career paths?
  - Is there a marginal benefit in funding research-oriented physicians or clinician-scientists? In other words: How many research-oriented clinicians or clinician-scientists can a university hospital "afford"?
- What role do medical organizations and professional associations play for *concrete CSP* with regard to the qualification or clinical and scientific further training of physicians and what lines of conflict or problems arise here (e.g. recognition of research periods in further specialist training)?
- Specifically, CSP* allows freedom for physicians employed and supported here. But does this special position also create a special pressure, e.g. to publish a lot, to acquire extensive third-party funding or similar?

**MANAGED CARE (10 MINUTES):**

Business management and competition are playing an increasingly important role in hospitals and thus inevitably also in university medicine. We would like to discuss with you whether this development is also relevant for *concrete CSP*.

How do you think the work of *concrete CSP* is influenced by these developments in healthcare?

- - Does *concrete CSP* fundamentally oppose the idea of competition by offering freedom to medical professionals, or do they accept it or modify it?
  - To what extent were changes towards competition and business management in health care taken into account when designing the *concrete CSP*?
  - What expectations are placed on *concrete CSP* by hospital management?
    - How do you deal with these expectations?
- How do you deal with the financing needs of *concrete CSP*, which arise especially in the context of continuations after the expiration of direct funding?
  - How can the sustainable establishment of *concrete CSP* be ensured in concrete terms?
    - Which developments in funding policy hinder the establishment of clinician scientist programs?

**PANDEMIC CONTROL (5 MINUTES):**

The COVID-19 pandemic has changed many processes in inpatient care and probably also in *concrete CSP*.

- What do you think were the most profound changes and what impact did this change have on the work *concrete CSP*?
- Do you think that the impact they mentioned on the work of *concrete CSP* will be permanent or will operations return to normal?
- Do you think that the pandemic has strengthened the importance of translation in general and therefore also the position of *concrete CSP*?
- What changes has the crisis brought about that will also influence the future work in research and clinical work in university medicine?

**END OF INTERVIEW (5 MINUTES):**

If we now arrived at the content of our conversation. Against this background:

- Is there anything we didn't ask but that you think is important if our concern is to understand the work of the *concrete CSP*?
- What do you think the role of clinician Scientist programs will be in the future for the development of translation, but also for careers in medicine?

Thank you, that brings us to the end of our interview! If you like, we would be happy to keep you updated on the results of our research.
