# Supplementary material for: What are the chances? Clinician scientist` career pathways in Germany
Source: BMC Med Educ. 2023 Sep 7;23:642. doi: 10.1186/s12909-023-04584-8 (PMC10486072; doi:10.1186/s12909-023-04584-8)
Supplement: Supplementary file 2 — Additional file 2: S table 1. Studied clinician scientist programs in Germany. To ensure anonymization, Table 1 is randomized. S table 2. Distribution of expert interviews per institution and level. [file 12909_2023_4584_MOESM2_ESM.docx]

S table 1: Studied clinician scientist programs in Germany. To ensure anonymization, Table 1 is randomized.

| **University /Medical Faculty at / Department** |
| --- |
| Ludwig-Maximilians-Universität München |
| Charité – Universitätsmedizin Berlin |
| Albert-Ludwigs-Universität Freiburg |
| Hannover Medical School |
| Mainz Research School of Translational Biomedicine (TransMed) |
| Julius-Maximilians-Universität Würzburg |
| Interdisziplinäres Zentrum für Klinische Forschung at Universitätsklinikum Jena |
| Universitätsmedizin Leipzig |
| Universität Regensburg |
| Universitätsklinikum Münster |
| Universitätsmedizin Rostock |
| Universitätsklinikum Schleswig-Holstein |
| Medizinische Fakultät OWL Bielefeld |

S table 2: Distribution of expert interviews per institution and level.

| **Number of studied CSP** | **Number of expert interviews** | **Number of interviews: management and politics** | **Number of interviews:**  **politics** | **Number of interviews: clinician scientist fellows** |
| --- | --- | --- | --- | --- |
| CSP 1 | 11 | 3 | 3 | 5 |
| CSP 2 | 3 | 1 |  | 2 |
| CSP 3 | 2 | 2 |  |  |
| CSP 4 | 2 | 2 |  |  |
| CSP 5 | 2 | 1 |  | 1 |
| CSP 6 | 4 | 1 |  | 3 |
| CSP 7 | 1 | 1 |  |  |
| CSP 8 | 1 | 1 |  |  |
| CSP 9 | 5 | 4 |  | 1 |
| CSP 10 | 1 | 1 |  |  |
| CSP 11 | 1 | 1 |  |  |
| CSP 12 | 2 | 2 |  |  |
| CSP 13 | 1 | 1 |  |  |
| **Total** | **36** | **21** | **3** | **12** |
